# Olanzapine-Induced Diabetic Ketoacidosis and Neuroleptic Malignant Syndrome: A Case Report

**DOI:** 10.7759/cureus.95327

**Published:** 2025-10-24

**Authors:** Motaz Almahmood, Amin Saied, Isra Eltazi, Bara M Al-Qudah, Abdulaziz Alalawi, Mohamed Zuhail K Peediyakkal, Abdulqadir J Nashwan

**Affiliations:** 1 Department of Internal Medicine, Hamad Medical Corporation, Doha, QAT; 2 Department of Neurology, Neurosciences Institute, Hamad Medical Corporation, Doha, QAT; 3 Department of Critical Care Medicine, Hamad Medical Corporation, Doha, QAT; 4 Department of Nursing and Midwifery Research, Hamad Medical Corporation, Doha, QAT

**Keywords:** antipsychotics, diabetic ketoacidosis, neuroleptic malignant syndrome, olanzapine, second-generation antipsychotics

## Abstract

With the growing use of second-generation antipsychotics, it is important to consider rare but serious side effects. One such complication is diabetic ketoacidosis (DKA), representing a severe form of metabolic derangement. Moreover, neuroleptic malignant syndrome (NMS) is a rare but potentially fatal idiosyncratic reaction linked to this category of medications. We report the case of a 69-year-old male, not diabetic, who developed both DKA and NMS, with olanzapine identified as a probable culprit. He was on a steady dose of olanzapine for three months. He had significant biochemical and clinical improvement following prompt discontinuation of medication and supportive care. DKA improved within 24 hours, and NMS improved within one week. To our knowledge, this remains a very rarely reported occurrence of olanzapine-induced coexisting DKA and NMS. Early detection and treatment of such cases, particularly in patients exposed to antipsychotics, is crucial.

## Introduction

Antipsychotics are considered first-line treatment for schizophrenia and a range of other psychiatric conditions [1]. Second-generation antipsychotics exert therapeutic effects through combined antagonism of dopamine D₂ and serotonin 5-HT₂A receptors, with additional activity at histamine, adrenergic, and muscarinic receptors contributing to their side-effect profile [2]. Nonetheless, adverse neurologic side effects have been reported with atypical antipsychotics, such as neuroleptic malignant syndrome (NMS), including olanzapine [1].

In 1960, “Syndrome malin des neuroleptiques” was the first description given for the constellation of symptoms of altered consciousness, muscle rigidity, hyperthermia, and autonomic dysfunction, and it was connected to the use of dopamine receptor blockers, with a focus on haloperidol effect [3]. An alternative theory proposed direct skeletal toxicity with chlorpromazine exposure in vitro, but without supportive clinical evidence [3]. Additionally, genetic susceptibility to calcium release, similar to malignant hyperthermia, has been associated with hyperthermia, rhabdomyolysis, and rigidity [3].

NMS is considered a diagnosis of exclusion; however, a high level of clinical suspicion is necessary for its accurate diagnosis. According to the Diagnostic and Statistical Manual of Mental Disorders: Fifth Edition (DSM-5), American Psychiatric Association, 2013, NMS is diagnosed when fever, muscle rigidity, and exposure to dopamine medication are present along with at least two associated signs, symptoms, or laboratory findings (diaphoresis, dysphagia, tremor, incontinence, altered level of consciousness, mutism, tachycardia, elevated or labile blood pressure, leukocytosis, elevated creatinine phosphokinase) that are not better explained by a substance-induced, neurological, or general medical condition [4].

Olanzapine, particularly among the young population, has been shown to impair glucose tolerance by reducing insulin sensitivity [5,6]. Research suggests that the WNT signaling pathway effector TCF7L2 has a crucial role in glucose regulation. Olanzapine-induced weight gain and reduced insulin sensitivity result in increased expression of TCF7L2 in the liver and skeletal muscle. Additionally, elevated insulin levels lead to higher TCF7L2 expression in adipose tissues. The rise in TCF7L2 expression across these tissues, which are all involved in glucose metabolism, suggests a potential mechanism for the metabolic dysfunction associated with olanzapine. This insight also highlights TCF7L2 as a potential therapeutic target for preventing or managing the adverse metabolic effects of olanzapine [7].

Hyperglycemic emergencies, including both diabetic ketoacidosis (DKA) and hyperosmolar hyperglycemic state, have been reported with second-generation antipsychotics, including olanzapine [8]. This case report aims to describe the clinical presentation, diagnostic reasoning, and management of a rare co-presentation of DKA and neuroleptic malignant syndrome NMS in an elderly patient after three months of stable olanzapine therapy. By documenting this delayed-onset presentation and its multifactorial context, we seek to highlight the importance of early recognition, appropriate differential diagnosis, and timely intervention in patients receiving second-generation antipsychotics.

## Case presentation

A 69-year-old gentleman was brought to our emergency department (ED) because he was unresponsive to sound or touch, reported by a caregiver. His baseline was walking with short steps, poor verbal communication, and dementia. He was under follow-up with the geriatric psychiatry team for behavioral disturbances secondary to Alzheimer’s dementia for six years, with a rapid decline in cognitive functions. The patient had not opened his bowels for two days before admission; however, there were no reported symptoms of nausea, vomiting, or any other complaints. There was no reported contact with sick patients.

Upon arrival at the ED, the patient had a decreased level of consciousness. He was on olanzapine 10 mg once a day at bedtime for three months before switching to mirtazapine 10 mg once a day at bedtime, memantine 10 mg twice daily (BID), and lorazepam 2 mg daily. The medication was taken once a day at bedtime, which was reduced one month before the presentation to 1 mg daily. The last investigations, conducted three months earlier, were within the normal range, including HbA1c at 5.2% (normal = <6%) and fasting glucose at 5.1 mmol/L (normal = 3.3 to 5.5). He was a retired professional football player with neither a smoking nor an alcohol history of trauma. He was married; however, given his advanced dementia, he had a private caregiver nurse and multiple admissions to a geriatrics facility with good family support.

When examined in the ED, he was tachypneic (36 breaths/minute), tachycardic (125 beats/minute), and febrile (39.4°C) with normal blood pressure (110/78 mmHg). He was 178 cm in height and weighed 70 kg with a body mass index (BMI) of 22.1 kg/m². The best response on the Glasgow Coma Scale (GCS) was 6/15. No focal neurological deficit was detected except rigidity in all limbs. Cardiovascular and respiratory examination showed sinus tachycardia, with no wheezes, crackles, or murmurs. Abdominal examination showed neither tenderness nor rebound tenderness. He had dry skin.

The patient was intubated due to a decreased level of consciousness. The initial laboratory investigations revealed marked metabolic derangements consistent with DKA and rhabdomyolysis (very high myoglobin and creatine phosphokinase (CPK), see Table 1). Table 1 summarizes the patient’s hematological, biochemical, and arterial blood gas findings on admission, along with their corresponding reference ranges. Notably, there was severe hyperglycemia, metabolic acidosis with an elevated anion gap and high ketones, B-hydroxybutyrate, acute kidney injury, and markedly elevated CPK and myoglobin levels, supporting the diagnoses of DKA and rhabdomyolysis.

**Table 1:** Laboratory results on admission with corresponding reference ranges.

Test	Result	Reference range
White blood cell count	16.8 × 10³/µL	4.0–11.0 × 10³/µL
Neutrophils	89%	40–70%
Hemoglobin	16.1 g/dL	13.5–17.5 g/dL
Hematocrit	55.0%	38–50%
Platelets	284 × 10³/µL	150–450 × 10³/µL
Plasma glucose	33.3 mmol/L	3.9–6.1 mmol/L
Blood urea nitrogen	20.5 mmol/L	2.5–7.1 mmol/L
Creatinine	396 µmol/L	62–106 µmol/L
Sodium	163 mmol/L	135–145 mmol/L
Potassium	2.7 mmol/L	3.5–5.0 mmol/L
Chloride	120 mmol/L	98–106 mmol/L
Total protein	79 g/L	60–80 g/L
Albumin	34 g/L	35–50 g/L
Aspartate aminotransferase	12 U/L	10–40 U/L
Alanine aminotransferase	23 U/L	7–56 U/L
Total bilirubin	7 µmol/L	3–21 µmol/L
Calcium	2.61 mmol/L	2.1–2.6 mmol/L
Phosphate	0.97 mmol/L	0.8–1.5 mmol/L
Creatine phosphokinase	>22,000 U/L	39–308 U/L
Myoglobin	17,251 ng/mL	<85 ng/mL
β-Hydroxybutyrate	4.97 mmol/L	0.03–0.30 mmol/L
Amylase	14 U/L	13–53 U/L
Lipase	37 U/L	13–60 U/L
Arterial pH	7.198	7.35–7.45
pCO₂	20 mmHg	35–45 mmHg
pO₂	77 mmHg	80–100 mmHg
HCO₃⁻	7.8 mmol/L	22–28 mmol/L
O₂ saturation	99%	95–100%
Anion gap	35.2 mmol/L	8–16 mmol/L
HbA1c	8.2%	<5.7%

**Figure 1:**
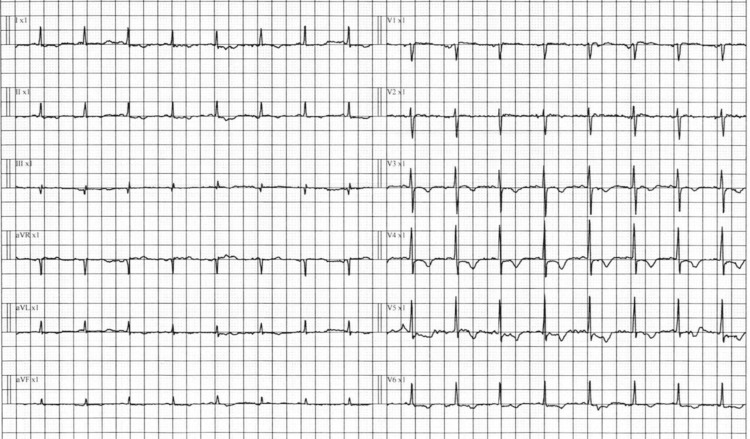
Electrocardiogram (ECG) findings. The 12-lead ECG shows sinus tachycardia with a heart rate >100 beats/minute. T-wave inversion is present in leads V3 through V6, suggestive of possible ischemic changes or strain pattern.

**Figure 2:**
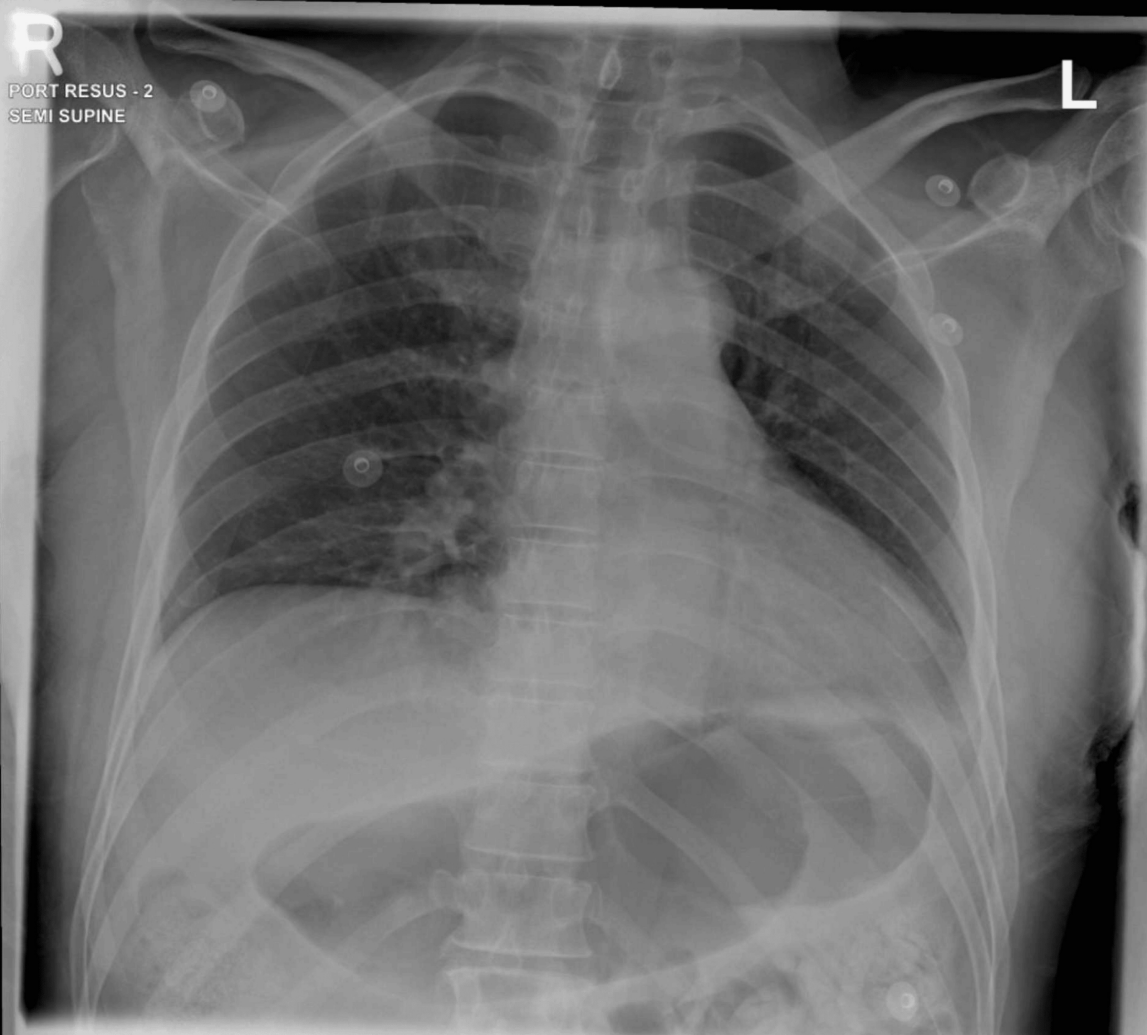
Chest X-ray findings. Portable semi-supine chest X-ray shows a left lower zone infiltrate with blunting of the left costophrenic angle, suggestive of left-sided pleural effusion or consolidation. There are prominent bronchovascular markings bilaterally. The stomach appears overdistended. No abnormal air-fluid levels are noted.

**Figure 3:**
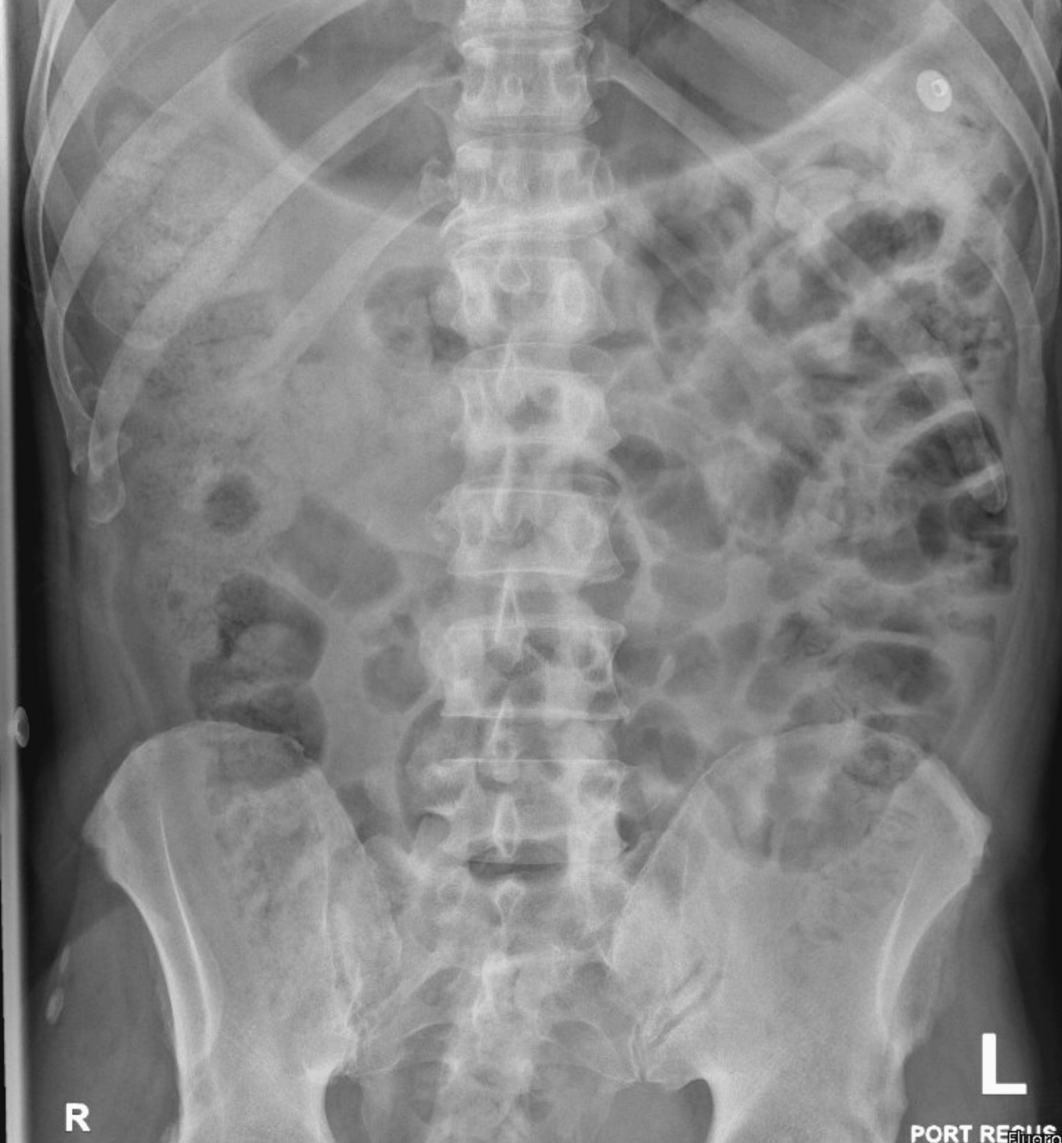
Abdominal X-ray findings. Supine abdominal X-ray demonstrates diffuse bowel distension with fecal loading consistent with fecal impaction. No evidence of abnormal air-fluid levels or free intraperitoneal air is seen.

Cerebrospinal fluid (CSF) analysis ruled out meningitis and encephalitis. Electrographically, he was in sinus tachycardia (Figure 1), with an unremarkable chest X-ray (Figure 2). Abdominal X-ray showed fecal impaction (Figure 3).

Hence, the patient’s working diagnoses were DKA and acute renal failure. In addition, the constellation of fever, muscle rigidity in all limb joints, bilateral knee jerk reflex, clonus on both feet, altered mental status, and rhabdomyolysis pointed toward NMS.

Although there was no hypotension, the patient showed severe dehydration induced by extreme hyperglycemia and metabolic acidosis. Fluids at 4 L/day were administered through intravenous infusion over six days. Close follow-up of volume status, urine output, rhabdomyolysis, renal parameters, and insulin was conducted via an infusion pump, according to local hospital guidelines.

Within 24 hours of insulin therapy, the plasma glucose level declined to less than 16 mmol/L, the anion gap level returned to the normal range, and the arterial blood gas analysis showed that he was no longer in an anion gap metabolic acidosis. On the second day of admission, given the clinical signs of NMS, the patient was started on dantrolene 80 mg BID for six days along with midazolam.

Infusion was administered at a rate of 1 µg/kg/minute for seven days, and amantadine 200 mg loading followed by 100 mg daily. Home medication, including olanzapine, mirtazapine, was already stopped at admission and not resumed. On the 10th day of admission, with fluid infusion of ≥2 L/day (from day seven to 10, before that was around 4 L daily), in addition to the treatment mentioned above, hyperthermia and rigidity improved, and the levels of CPK and creatinine decreased to 15,487 U/L and 208 μmol/L, respectively. Moreover, the myoglobin level was 3,760 ng/mL. His urine output improved, and both creatinine and CPK concentrations returned to normal levels.

Regarding GCS and consciousness, it was challenging to assess acutely because the patient was mechanically ventilated and required assistance. He had regained consciousness, as evidenced by spontaneous eye opening and limb movements. Speech could not be assessed. The patient was transferred to a rehabilitation facility after approximately 24 days of admission. Later on, for dementia and behavioral issues, he was on memantine 10 mg tablet twice a day, mirtazapine 7.5 mg once a day, rivastigmine transdermal patch 4.6 mg/24 hours, but never on olanzapine again. This presentation did not recur during the one-year follow-up period.

With long-term follow-up (10 months after the acute event), it was documented that the patient is now in a geriatric long-term facility, showing improvement in consciousness and mobilizing with a walker, assisted by two people during physiotherapy sessions

## Discussion

Olanzapine is a widely used atypical antipsychotic that is very effective in treating schizophrenia, schizoaffective disorder, and other psychotic conditions [9]. Atypical antipsychotics have been shown to carry an increased risk of metabolic diseases such as obesity, glucose intolerance, and diabetes [10]. NMS is a serious adverse effect in psychosis treatment, characterized by autonomic nervous system instability with high fever, extrapyramidal symptoms such as muscle rigidity, and alterations in consciousness, including confusion and coma [11]. NMS is an uncommon yet potentially fatal adverse reaction to antipsychotic drugs [12]. This discussion explores the dual occurrence of olanzapine-induced DKA and NMS, drawing on similar cases from the literature.

NMS is more frequently seen in young male patients, especially when symptoms such as agitation are present [13]. Physicians need to be alert to recognizing patients who are more susceptible to developing NMS. Factors such as male gender, agitation, physical exhaustion, dehydration, and neurological abnormalities are linked to a higher risk of NMS [11]. NMS is more frequently observed with typical antipsychotic treatments. However, it can also occur with atypical antipsychotics such as olanzapine, though this syndrome is rare with these medications. It has been proposed that the capacity of neuroleptics to induce NMS is linked to their ability to block dopamine in the nigrostriatal pathway, mesocortical pathway, and hypothalamic nucleus [13].

Furthermore, NMS can develop at any time after taking dopamine antagonists. This condition may also arise in patients who abruptly stop using dopamine agonists [10]. Our patient had risk factors that predisposed him to this syndrome. Male patients are twice as likely to develop this syndrome compared to females, especially when factors such as dehydration and agitation are present [12]. In our case, clinical findings included muscle rigidity, hyperreflexia, stiffness, reduced level of consciousness, rhabdomyolysis, and fever, along with laboratory tests (such as CPK of >22,000 U/L), after exposure to the causative agent. These symptoms could not be attributed to other diseases or medications, and discontinuation of the drug led to significant clinical improvement. The clinical presentation and laboratory findings were characteristic of NMS, making it unnecessary to rechallenge with the drug. According to the World Health Organization-Uppsala Monitoring Centre causality assessment system, olanzapine is classified as “probable” or “likely” to be the cause [14].

The clinical presentation of DKA is frequently the earliest indication of diabetes mellitus caused by antipsychotic drugs [15]. The exact mechanism behind olanzapine-induced impairment of insulin secretion remains unknown. However, it is suspected to involve disrupted insulin secretion and a failure in the compensatory enhancement of β-cell function via the neuropancreatic axis [16-18]. Fertig et al. were the first to suggest a link between olanzapine and abnormal glucose metabolism in 1998. Later, in 2003, Wilson et al. documented cases of DKA associated with atypical antipsychotics [16-18]. According to existing reports, the incidence of DKA following the administration of atypical antipsychotic medications primarily occurs in the early stages of treatment. One report indicated that DKA typically developed around 5.8 weeks after starting the medication. However, as in our case, there have been instances of long-term administration lasting one year or more. Wehring et al. reported deaths occurring at 14.5, 25.5, and 59.5 months due to DKA following clozapine administration [19].

In this case, as we identified a link to the medication, we stopped administering the drug and initiated intensive glycemic control using fluids and insulin. We also started benzodiazepine infusion, dantrolene [20], and amantadine [21] to address NMS-related, rhabdomyolysis-induced acute renal failure. The patient had improvement in plasma glucose, anion gap, creatinine, and CPK levels after these treatments. Even though neurological side effects are rare with atypical antipsychotics, their incidence should be closely monitored. This involves tracking CPK levels when administering atypical antipsychotics such as olanzapine [22].

## Conclusions

This case highlights the importance of recognizing the potential for severe and simultaneous complications associated with atypical antipsychotics. Atypical antipsychotics, including olanzapine, may contribute to acute metabolic and neuromuscular crises in vulnerable older adults, yet such presentations are often multifactorial. Prompt recognition and protocol-guided DKA management with rigorous potassium control, alongside NMS-directed therapy, are essential for favorable outcomes. The coexistence of DKA and NMS in a non-diabetic elderly patient illustrates the need for heightened clinical vigilance, even in individuals with previously standard glycemic profiles. Clinicians should be aware that atypical antipsychotics such as olanzapine can precipitate acute metabolic and neurological crises, including rhabdomyolysis and acute renal failure. Early identification, prompt withdrawal of the offending agent, and aggressive supportive management, including IV fluid and DKA and NMS management, remain critical for favorable outcomes. Reporting such rare and complex presentations contributes to the growing body of knowledge and may aid in developing more effective strategies for monitoring, prevention, and early intervention in vulnerable populations.
